# Supplementary material for: Basic fibroblast growth factor helps protect facial nerve cells in a freeze-induced paralysis model
Source: PLoS One. 2025 Feb 6;20(2):e0312357. doi: 10.1371/journal.pone.0312357 (PMC11801600; doi:10.1371/journal.pone.0312357)
Supplement: S1 Table — (PDF) [file pone.0312357.s001.pdf]

Control group

	No.	right	left	Facial neuron mortality rate
	**1**	4378	4148	5.3
	**2**	4483	4126	8
	**3**	3626	3443	5
**1W**	**4**	4517	4648	-2.9
	**5**	3606	3493	3.1
	**6**	3782	3702	2.1
	**7**	4017	3976	1
	**Ave.**	4058	3934	**3.1**
	**1**	4870	3552	27.1
	**2**	4213	3465	17.8
	**3**	4735	3426	27.6
**4W**	**4**	5017	4196	16.4
	**5**	4812	3712	22.9
	**6**	4585	3689	19.5
	**7**	4508	3769	16.4
	**Ave.**	4677	3687	**21.1**
	**1**	4204	3017	28.2
	**2**	4470	3170	29.1
	**3**	4878	3274	32.9
**10W**	**4**	4626	3335	27.9
	**5**	4630	3413	26.3
	**6**	5222	3535	32.3
	**7**	4474	3283	26.6
	**Ave.**	4643	3290	**29.0**

bFGF group

	No.	right	left	Facial neuron mortality rate
	**1**	4233	4073	3.8
	**2**	4421	4371	1.1
	**3**	3831	3704	3.3
**1W**	**4**	3691	3614	2.1
	**5**	3530	3471	1.7
	**6**	3933	3856	2

	**7**	3756	3624	3.5
	**Ave.**	3914	3816	**2.5**
	**1**	4217	3622	14.1
	**2**	4213	3461	17.9
	**3**	5139	4578	10.9
**4W**	**4**	4904	4261	13.1
	**5**	4719	4080	13.5
	**6**	4520	3793	16.1
	**7**	4491	3927	12.6
	**Ave.**	4600	3960	**14.0**
	**1**	4135	3452	16.5
	**2**	4670	4030	13.7
	**3**	4513	3930	12.9
**10W**	**4**	4725	4008	15.2
	**5**	4480	3644	18.7
	**6**	4336	3638	16.1
	**7**	4382	3615	17.5
	**Ave.**	4463	3760	**15.8**
