# Supplementary material for: Basic fibroblast growth factor helps protect facial nerve cells in a freeze-induced paralysis model
Source: PLoS One. 2025 Feb 6;20(2):e0312357. doi: 10.1371/journal.pone.0312357 (PMC11801600; doi:10.1371/journal.pone.0312357)
Supplement: S2 Table — (PDF) [file pone.0312357.s002.pdf]

Control	Week1			
Number	eyelid	nose	lip	sum
1	0	0	0	0
2	0	0	0	0
3	0	0	0	0
4	0	0	0	0
5	0	0	0	0
6	0	0	0	0
7	0	0	0	0
	Week2			
	eyelid	nose	lip	sum
1	0	0	0	0
2	0	0	0	0
3	0	0	0	0
4	0	0	0	0
5	0	0	0	0
6	0	0	0	0
7	0	0	0	0
	Week3			
	eyelid	nose	lip	sum
1	0	0	0	0
2	0	0	0	0
3	1	0	0	1
4	0	0	0	0
5	0	0	0	0
6	0	0	0	0
7	0	0	0	0
	Week4			
	eyelid	nose	lip	sum
1	1	0	0	1
2	1	0	0	1
3	1	1	0	2
4	0	1	0	1
5	1	0	0	1

6	1	1	0	2
7	1	0	0	1

Week5

	eyelid	nose	lip	sum
1	2	1	0	3
2	1	0	1	2
3	1	1	0	2
4	0	1	0	1
5	1	1	0	2
6	2	1	0	3
7	1	0	0	1

Week6

	eyelid	nose	lip	sum
1	2	1	0	3
2	2	0	1	3
3	1	1	0	2
4	1	1	0	2
5	2	1	0	3
6	2	2	1	5
7	1	0	1	2

Week7

	eyelid	nose	lip	sum
1	2	2	0	4
2	2	1	1	4
3	2	1	1	4
4	1	1	1	3
5	2	1	1	4
6	2	2	1	5
7	1	1	1	3

Week8

	eyelid	nose	lip	sum
1	2	2	1	5
2	2	2	1	5

3	2	1	2	5
4	2	1	1	4
5	2	1	1	4
6	2	2	1	5
7	2	1	1	4

Week9

	eyelid	nose	lip	sum
1	2	2	2	6
2	2	2	1	5
3	2	2	2	6
4	2	1	1	4
5	2	1	2	5
6	2	2	2	6
7	2	1	2	5

Week10

	eyelid	nose	lip	sum
1	2	2	2	6
2	2	2	1	5
3	2	2	2	6
4	2	2	1	5
5	2	1	2	5
6	2	2	2	6
7	2	1	2	5
