# Supplementary material for: Basic fibroblast growth factor helps protect facial nerve cells in a freeze-induced paralysis model
Source: PLoS One. 2025 Feb 6;20(2):e0312357. doi: 10.1371/journal.pone.0312357 (PMC11801600; doi:10.1371/journal.pone.0312357)
Supplement: S3 Table — (PDF) [file pone.0312357.s003.pdf]

Control

Week1

Number	eyelid	nose	lip	sum
1	0	0	0	0
2	0	0	0	0
3	0	0	0	0
4	0	0	0	0
5	0	0	0	0
6	0	0	0	0
7	0	0	0	0

Week2

	eyelid	nose	lip	sum
1	0	0	0	0
2	0	0	0	0
3	0	0	0	0
4	0	0	0	0
5	0	0	0	0
6	0	0	0	0
7	0	0	0	0

Week3

	eyelid	nose	lip	sum
1	0	0	0	0
2	0	0	0	0
3	0	0	0	0
4	0	0	0	0
5	0	0	0	0
6	0	0	0	0
7	0	0	0	0

Week4

	eyelid	nose	lip	sum
1	1	0	0	1
2	0	0	0	0
3	0	0	0	0
4	0	0	0	0
5	1	0	0	1

6	1	0	0	1
7	1	0	0	1

Week5

	eyelid	nose	lip	sum
1	1	0	0	1
2	1	0	0	1
3	0	0	0	0
4	0	0	0	0
5	1	0	0	1
6	2	0	0	2
7	1	0	0	1

Week6

	eyelid	nose	lip	sum
1	1	0	0	1
2	1	0	1	2
3	0	0	0	0
4	1	0	0	1
5	1	0	1	2
6	2	1	0	3
7	1	1	0	2

Week7

	eyelid	nose	lip	sum
1	2	0	0	2
2	1	1	1	3
3	1	1	0	2
4	1	1	0	2
5	2	1	1	4
6	2	1	0	3
7	2	1	0	3

Week8

	eyelid	nose	lip	sum
1	2	0	1	3
2	2	1	1	4

3	1	2	0	3
4	1	1	0	2
5	2	1	1	4
6	2	1	0	3
7	2	1	0	3

Week9

	eyelid	nose	lip	sum
1	2	2	2	6
2	2	1	1	4
3	1	2	0	3
4	1	1	1	3
5	2	1	1	4
6	1	1	0	2
7	2	2	1	5

Week10

	eyelid	nose	lip	sum
1	2	2	2	6
2	2	1	1	4
3	1	2	0	3
4	1	1	2	4
5	2	2	1	5
6	2	1	1	4
7	2	2	1	5
